# Nesprin-2-dependent ERK1/2 compartmentalisation regulates the DNA damage response in vascular smooth muscle cell ageing

**DOI:** 10.1038/cdd.2015.12

**Published:** 2015-03-06

**Authors:** D T Warren, T Tajsic, L J Porter, R M Minaisah, A Cobb, A Jacob, D Rajgor, Q P Zhang, C M Shanahan

**Affiliations:** 1 British Heart Foundation Centre of Research Excellence, Cardiovascular Division, King's College London, London SE5 9NU, UK; 2 Department of Medicine, Addenbrooke's Hospital, Cambridge CB2 2QQ, UK

## Abstract

Prelamin A accumulation and persistent DNA damage response (DDR) are hallmarks of vascular smooth muscle cell (VSMC) ageing and dysfunction. Although prelamin A is proposed to interfere with DNA repair, our understanding of the crosstalk between prelamin A and the repair process remains limited. The extracellular signal-regulated kinases 1 and 2 (ERK1/2) have emerged as key players in the DDR and are known to enhance ataxia telangiectasia-mutated protein (ATM) activity at DNA lesions, and in this study, we identified a novel relationship between prelamin A accumulation and ERK1/2 nuclear compartmentalisation during VSMC ageing. We show both prelamin A accumulation and increased DNA damage occur concomitantly, before VSMC replicative senescence, and induce the localisation of ERK1/2 to promyelocytic leukaemia protein nuclear bodies (PML NBs) at the sites of DNA damage via nesprin-2 and lamin A interactions. Importantly, VSMCs treated with DNA damaging agents also displayed prelamin A accumulation and ERK compartmentalisation at PML NBs, suggesting that prelamin A and nesprin-2 are novel components of the DDR. In support of this, disruption of ERK compartmentalisation at PML NBs, by either depletion of nesprin-2 or lamins A/C, resulted in the loss of ATM from DNA lesions. However, ATM signalling and DNA repair remained intact after lamins A/C depletion, whereas nesprin-2 disruption ablated downstream Chk2 activation and induced genomic instability. We conclude that lamins A/C and PML act as scaffolds to organise DNA-repair foci and compartmentalise nesprin-2/ERK signalling. However, nesprin-2/ERK signalling fidelity, but not their compartmentalisation at PML NBs, is essential for efficient DDR in VSMCs.

DNA damage is a major driving force during cellular ageing, and it has been implicated in hastening the development of cardiovascular diseases, including atherosclerosis where the accumulation of senescent cells has been shown to accelerate disease.^1^ Normally, DNA damage is efficiently repaired by the DNA damage response (DDR), a complex signalling cascade of proteins that include sensors (NBS1/MRE11), transducers (ataxia telangiectasia-mutated protein (ATM)/ataxia telangiectasia- and Rad3-related protein (ATR)) and effectors (p53/p21). However, if damage is overwhelming or repair is inefficient, the accumulation of unrepaired DNA damage leads to persistent DNA damage signalling and premature senescence.^2^ Recently, the nuclear lamina has been implicated in the DDR, and its disruption is associated with accelerated cardiovascular ageing.^3^

The nuclear lamina is composed of A-type (lamins A/C) and B-type (lamins B1/B2) lamins that underlie the nuclear envelope (NE) and extend throughout the nucleoplasm^4^ to form a scaffold that is essential for the compartmentalisation and the integrity of nuclear signalling.^4^ The pathological accumulation of lamin A precursors such as prelamin A or progerin causes Hutchinson Gilford Progeria Syndrome (HGPS), a disease of accelerated ageing where patients develop severe early-onset arteriosclerosis characterised by vascular smooth muscle cell (VSMC) attrition.^4^ During the DDR, lamin A interacts with Ku70 and *γ*H2AX, and forms a framework that is essential for the positional stability of repair foci.^5, 6^ Prelamin A accumulation interferes with DNA-repair processes and has been shown to delay the recruitment of DNA-repair proteins such as 53BP1 to double strand breaks (DSBs)^7^ and cause mislocalisation of DNA PK_cs_.^8^ Fibroblasts and induced pluripotent stem (iPS) cells derived from HGPS patients show persistent DNA damage signalling and premature senescence.^8^ Importantly, aged VSMCs, both *in vitro* and *in vivo*, also accumulate prelamin A and activate persistent DDR, suggesting a key role for the nuclear lamina in vascular ageing.^2^

The nesprin family of spectrin-repeat (SR) proteins were first identified as NE lamin A-binding proteins. However, both nesprin-1 and nesprin-2 show extensive alternate splicing, and variants have been shown to localise to multiple nuclear and cytoplasmic compartments.^9^ One such variant, nesprin-2*β*ΔKASH1, retains a lamin A-binding region and localises to promyelocytic leukaemia protein nuclear bodies (PML NBs) in VSMCs.^10^ Previously, we demonstrated that nesprin-2*β*ΔKASH1 scaffolds extracellular signal-regulated kinases 1 and 2 (ERK1/2) at PML NBs and acts to regulate nuclear ERK1/2 activity and downstream VSMC proliferation.^10^ Importantly, both ERK and PML NBs have also been implicated in the DDR; PML and ERK1/2 localise at DNA lesions where ERK1/2 enhance ATM- and ATR-mediated repair.^11, 12, 13^ In addition, PML and ERK1/2 are essential for regulating the cell cycle in response to DNA damage; PML forms nucleolar cap structures that sequester MDM2 and activate DNA damage-mediated p53 signalling, while ERK1/2 are essential for efficient G2/M checkpoint activation.^14, 15, 16^ Similar to nesprin-2, PML and ERK1/2 have also been shown to associate with the nuclear lamina suggesting that the nuclear lamina, potentially via nesprin-2*β*ΔKASH1, may regulate ERK compartmentalisation during the DDR.^17, 18^

In this study, we identify a novel signalling complex that regulates compartmentalisation of ERK1/2 during the DDR in VSMCs. We show that the nuclear lamina tethers PML NBs and spatially organises nuclear signalling events. Disruption of this organisation results in ATM mislocalisation from DNA-repair foci and impairs downstream DNA-repair signalling, ultimately leading to genomic instability.

## Results

### Prelamin A accumulation, persistent DDR and increased ERK1/2 compartmentalisation at PML NBs are associated with VSMC ageing

**Figure 1:**
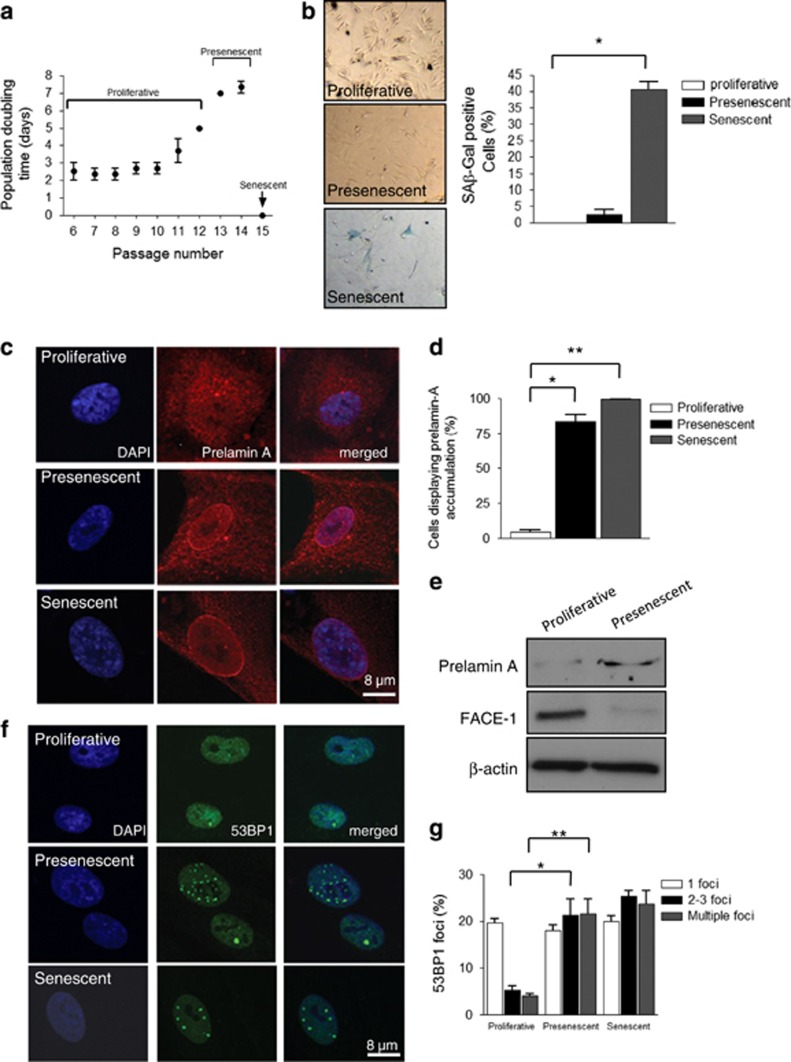
Characterisation of VSMC ageing. (**a**) VSMCs grown *in vitro* display three distinct growth phases we termed proliferative (PDT 2–5 days), presenescence (PDT 6–8 days) and senescence. Graph shows the combined growth curve data from three independent experiments. (**b**) Cells from each phase stained for SA-*β*-gal activity; graph shows the combined data of three independent experiments (*P=0.0033). (**c**) IF images of prelamin A accumulation (red) and DAPI (blue). (**d**) Quantification of prelamin A accumulation in presenescent (*P=0.0051) and senescent (**P=0.0001) VSMCs. (**e**) WB of prelamin A and FACE1 levels in proliferative and presenescent VSMCs. (**f**) IF images of 53BP1 (green), a marker of persistent DNA damage signalling, and DAPI (blue). (**g**) Quantification of cells displaying 53BP1 foci in presenescent (*P=0.0263) and senescent (**P=0.0328) cells. Graphs represent the combined data from three independent experiments counting 300 cells per condition

To model ageing, VSMCs were serially passaged *in vitro*, which identified three distinct phases of cell growth. Early-passage cells proliferated with a population-doubling time (PDT) of 2–5 days showed no evidence of DNA damage, measured by formation of *γ*H2AX and 53BP1 foci, and did not accumulate prelamin A (Figures 1a–g and Supplementary Figures 1 and 2). After a number of further passages dependent on cell isolate, VSMCs reduced their growth rate (PDT of 6–8 days) and entered presenescence, characterised by increased prelamin A levels and persistent DNA damage signalling marked by increased *γ*H2AX and 53BP1 foci (Figures 1a–g and Supplementary Figures 1 and 2). Western blotting revealed that decreased FACE1 levels were associated with prelamin A accumulation in presenescent VSMCs (Figure 1e). However, these cells did not show increased senescence-associated *β*-galactosidase activity (SA-*β*-gal; Figure 1b and Supplementary Figure 1). This contrasted with senescent VSMCs that completely ceased proliferation and stained strongly for SA-*β*-gal, prelamin A, and also showed increased 53BP1 foci (Figures 1a–g and Supplementary Figures 1 and 2).^2^

**Figure 2:**
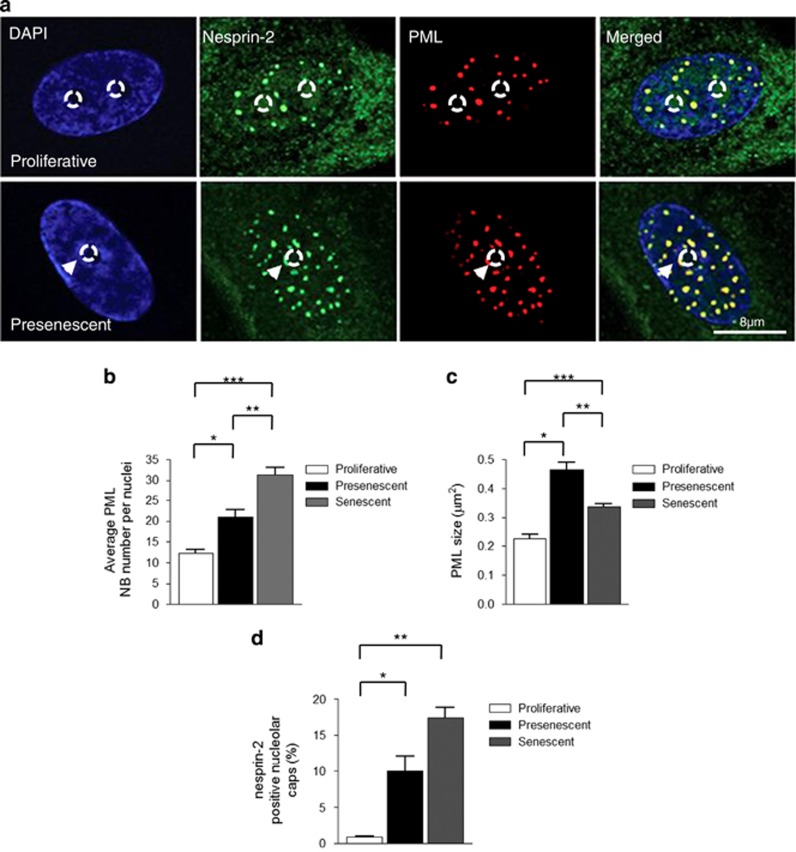
PML NB reorganisation during VSMC ageing. (**a**) IF images of nesprin-2 and PML NBs in proliferative and presenescent VSMCs; DAPI (blue), nesprin-2 (green) and PML (red). Arrowheads mark nucleolar caps. Circle marks nucleoli position. Quantification of (**b**) average number of PML NBs per nucleus (*P=0.0339, **P=0.01, ***P=0.001); (**c**) average PML NB size (*P=<0.001, **P=<0.001, ***P=<0.001); and (**d**) number of cells displaying nucleolar caps (*P=0.0116 and **P=0.0076) in proliferative, presenescent and senescent phases of growth. Graphs show the combined data of three independent experiments counting 300 cells

**Figure 3:**
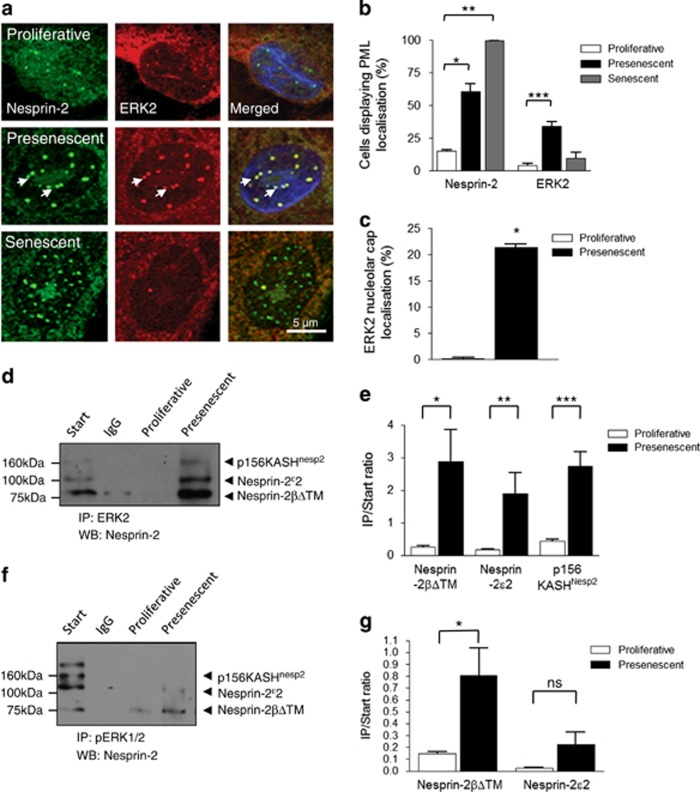
Increased nesprin-2/ERK1/2 compartmentalisation at PML NBs during VSMC ageing. (**a**) IF showing the localisation of nesprin-2 (green) and ERK2 (red) in proliferative, presenescent and senescent VSMCs. Arrows mark nucleolar caps. Quantification of (**b**) cells displaying nesprin-2 (*P=0.0114 and **P=0.0004) and ERK2 (***P=0.0117) localisation at PML NBs; and (**c**) proliferative and presenescent VSMCs displaying ERK2-positive nucleolar caps (*P=0.0008). Graphs show the combined data of three independent experiments counting 300 cells. IPs showing the association between (**d**) ERK2 and (**f**) pERK1/2 with nesprin-2 in proliferative and presenescent VSMCs. Graphs show the combined data of three independent experiments performing densitometry measurement of precipitated/start ratio for each nesprin variant precipitated by (**e**) ERK2 (*P=0.025, **P=0.0226, ***P=0.0004) and (**g**) pERK1/2 (*P=0.0458)

Immunofluorescence microscopy (IF) revealed that presenescent and senescent VSMCs contained increased numbers of PML NBs per nuclei compared with proliferative VSMCs (Figures 2a and b, and Supplementary Figure 2). Further analysis revealed that presenescent VSMCs contained larger PML NBs than their proliferative and senescent counterparts, suggesting PML NBs are remodelled during VSMC ageing (Figures 2a–c and Supplementary Figure 2). Nesprin-2 co-localised with PML NBs (Figure 2a), and in a small subpopulation of VSMCs, both PML and nesprin-2 decorated the nucleoli and formed nucleolar cap-like structures (Figures 2a and d), with the frequency of this nucleolar localisation significantly increased in presenescent and senescent cultures (Figures 2a and d). ERK localisation also differed between proliferative, presenescent and senescent VSMCs. In proliferative cells, ERK2 nuclear staining was diffuse and co-localisation with nesprin-2 at PML NBs was weak. In contrast, presenescent cells displayed strong nesprin-2 and ERK2 co-localisation at PML NBs that decorated the nucleolar periphery (Figures 3a–c and Supplementary Figure 3). Senescent VSMC PML NBs retained their association with nesprin-2, but ERK2 staining was diffuse with reduced co-localisation at PML NBs (Figures 3a and b, and Supplementary Figure 3) suggesting the ERK/nesprin-2/PML complex was specific to presenescent VSMCs. Immunoprecipitation (IP) confirmed increased interactions between total ERK2, active ERK1/2 and nesprin-2 in presenescent VSMCs compared with proliferative VSMCs (Figures 3d and e) Moreover, ERK2 precipitated nesprin-2*β*ΔKASH1 (75 kDa) variant and two larger previously identified NE nesprin-2 variants, whereas pERK1/2 antibodies predominantly precipitated the nesprin-2*β*ΔKASH1 (75 kDa) variant (Figures 3d and g, and Supplementary Figure 4). Importantly, ERK2 and pERK1/2 failed to precipitate nesprin-2*β*ΔKASH1 or the larger nesprin-2 variants from senescent VSMCs, further suggesting that ERK2 is lost from the complex (Supplementary Figure 5).

The stabilization of the complex in presenescent VSMCs suggested that it may have a role in DNA damage signalling. Western blot (WB) confirmed increased levels of *γ*H2AX in presenescent VSMCs (Supplementary Figure 6). Co-localisation with *γ*H_2_AX foci (marking double strand DNA breaks) revealed that PML-associated ERK2 abutted the sites of DSBs as shown by the close juxtaposition of nesprin-2 and ERK2 (Supplementary Figure 6).

### Lamins A/C are essential for nesprin-2/ERK compartmentalisation at PML NBs

**Figure 4:**
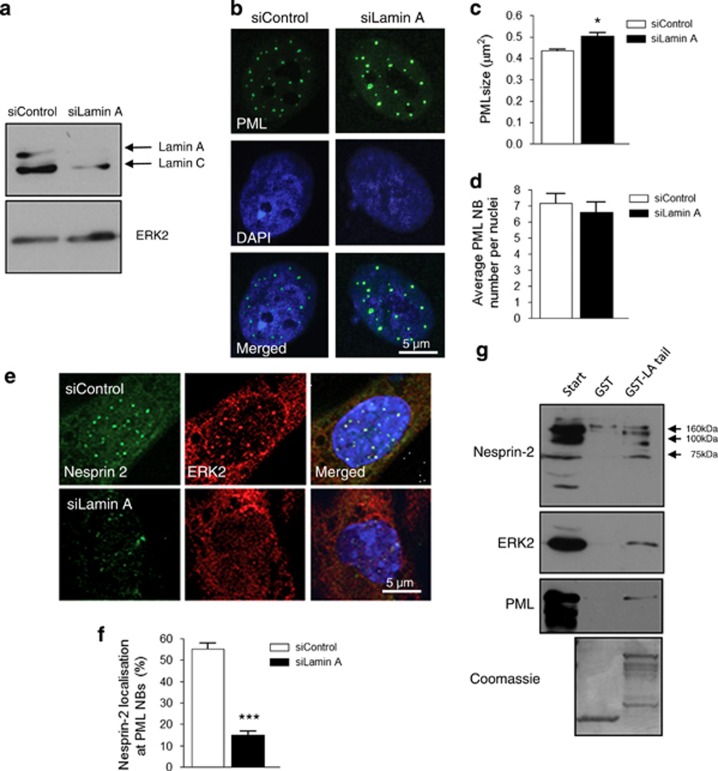
The A-type lamins are essential for nesprin-2/ERK/PML complex organisation in VSMCs. (**a**) WB of control and lamin A-depleted VSMC lysates. (**b**) IF showing PML organisation (green) and DAPI (blue) in control and lamin A-depleted VSMCs; Quantification of (**c**) PML size (*P=<0.0001) and (**d**) PML number per nuclei in control and lamin A-depleted VSMCs. Graphs show the combined data of three independent experiments counting 300 cells. (**e**) IF showing nesprin-2 (green) and ERK2 (red) localisations in control and lamin A-depleted nuclei. (**f**) Quantification of nesprin-2 localisation at PML NBs in control and lamin A-depleted VSMC nuclei (***P=0.0009). Graph shows the combined data of three independent experiments counting 300 cells. (**g**) WB of GST-pull downs using bacterial expressed lamin A tail construct and VSMC lysates

To investigate whether an intact nuclear lamina was necessary for complex formation, lamin A/C was depleted from VSMCs using siRNA (Figure 4a). Although the number of PML NBs per nuclei remained unaltered, they were larger in lamin A/C-depleted VSMCs compared with control (Figures 4b–d). Moreover, lamin A/C-depleted cells showed reduced nesprin-2 and ERK2 localisation at PML NBs (Figures 4e and f). To determine the long-term impact of lamin A/C depletion on VSMC proliferation, cells were subjected to three rounds of siRNA-mediated knockdown. Extended lamin A depletion had no effect on 53BP1 foci number per nuclei, PDT or SA-*β*-gal staining (Supplementary Figure 7). GST-pull down assays using a construct containing the C-terminal portion of lamin-A, which consists of an immunoglobulin domain-like region precipitated nesprin-2*β*ΔKASH1, ERK2 and PML, and this was not observed with GST alone (Figure 4g), suggesting the complex could bind directly to lamin A. Several other nesprin-2 variants were also efficiently precipitated as expected, including nesprin-2*ɛ*2 and p156KASH^nesp2^ (Figure 4g and Supplementary Figure 4).

### Prelamin A-induced DNA damage triggers ERK1/2 compartmentalisation at DNA lesions

**Figure 5:**
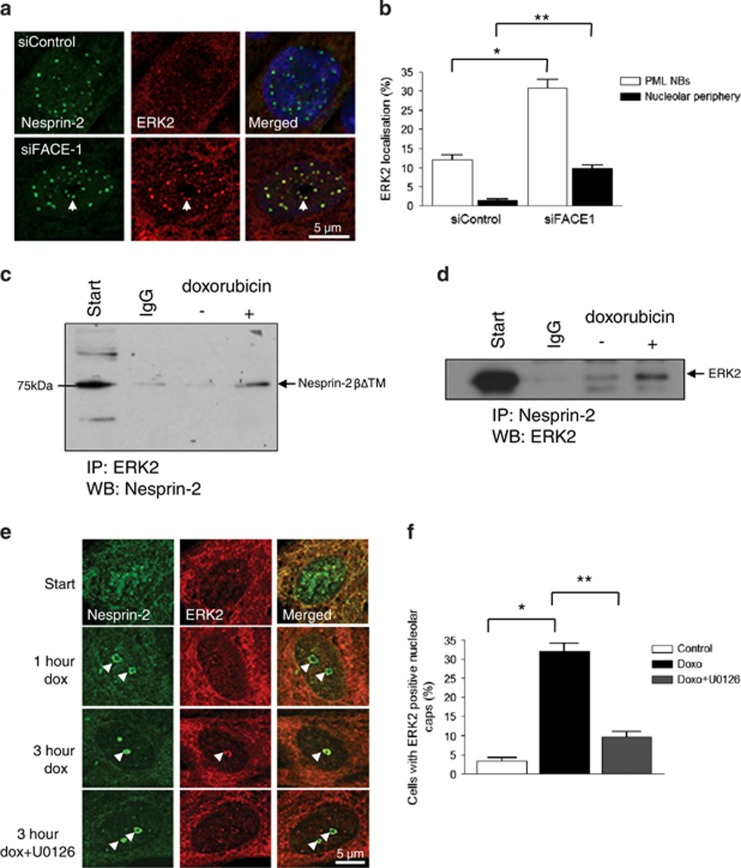
Prelamin A accumulation induces ERK1/2 compartmentalisation at PML NBs that abut DNA lesions. (**a**) IF showing nesprin-2 (green) and ERK2 (red) localisations in control and FACE1-depleted nuclei. Arrows mark PML NBs localised towards the nucleolar periphery. (**b**) Quantification of ERK2 localisation at PML NBs (*P=0.0278) and nucleolar periphery (**P=0.0202) in control and FACE1-depleted nuclei. Graph shows the combined data of three independent experiments counting 300 cells. IP of (**c**) ERK2 antibody to pellet 75 kDa nesprin-2*β*ΔKASH1 variant and (**d**) nesprin-2 antibody to pellet ERK2 from control and doxorubicin-treated VSMCs. (**e**) IF of doxorubicin-treated proliferative VSMCs; nesprin-2 (green), ERK2 (red). Arrowheads mark nucleolar caps. (**f**) Quantification of cells displaying ERK2-positive nucleolar caps after a 3-h doxorubicin treatment (*P=0.0081 and **P=0.0146). Graph shows the combined data of three independent experiments counting 300 cells

Next, we investigated whether nuclear lamina disruption induced by prelamin A was the triggering event for ERK1/2 compartmentalisation at PML NBs. SiRNA-mediated knockdown of the lamin A processing the enzyme FACE1 to induce prelamin A accumulation in proliferative VSMCs (Supplementary Figure 8) triggered an increase in cells with ERK2 localisation at PML NBs (Figures 5a and b). No nucleolar cap formation was observed; however, ERK2-associated PML NBs did redistribute toward the nucleolar periphery in FACE1-depleted cells (Figures 5a and b). As prelamin A accumulation promotes DNA damage, we also investigated whether DNA damage *per se* could induce complex formation. Treatment of proliferative VSMCs with the DNA damage inducer doxorubicin increased the levels of phosphorylated ERK1/2 in VSMC lysates (Supplementary Figure 9), and the association between nesprin-2*β*ΔTM and both ERK2 and phosphorylated ERK1/2 was also enhanced (Figures 5c and d, and Supplementary Figure 9). Subcellular fractionation and WB showed that prelamin A also accumulated in the nuclear fraction in response to doxorubicin (Supplementary Figure 9) suggesting there is a potential feedback between ERK compartmentalisation and prelamin A accumulation in response to DNA damage.

Confocal immunofluorescence showed that doxorubicin treatment caused a dose-dependent, relocalisation of both nesprin-2 and PML into nucleolar cap structures (Figures 5e and f, and Supplementary Figure 9). After a short-term treatment with doxorubicin (1 h), ERK2 was lacking in these nucleolar caps (Figures 5e and f). However, prolonged doxorubicin (3 h) treatment enhanced the association of ERK2 with the nesprin-2-positive nucleolar caps (Figures 5e and f). Pretreatment with the MEK inhibitor U0126 failed to disrupt nesprin-2 reorganisation into the nucleolar cap, but it dramatically reduced ERK2 association with the nucleolar cap suggesting that active ERK1/2 are components of this complex (Figures 5e and f).

### Complex formation is essential for ATM signalling fidelity and its disruption leads to genomic instability

**Figure 6:**
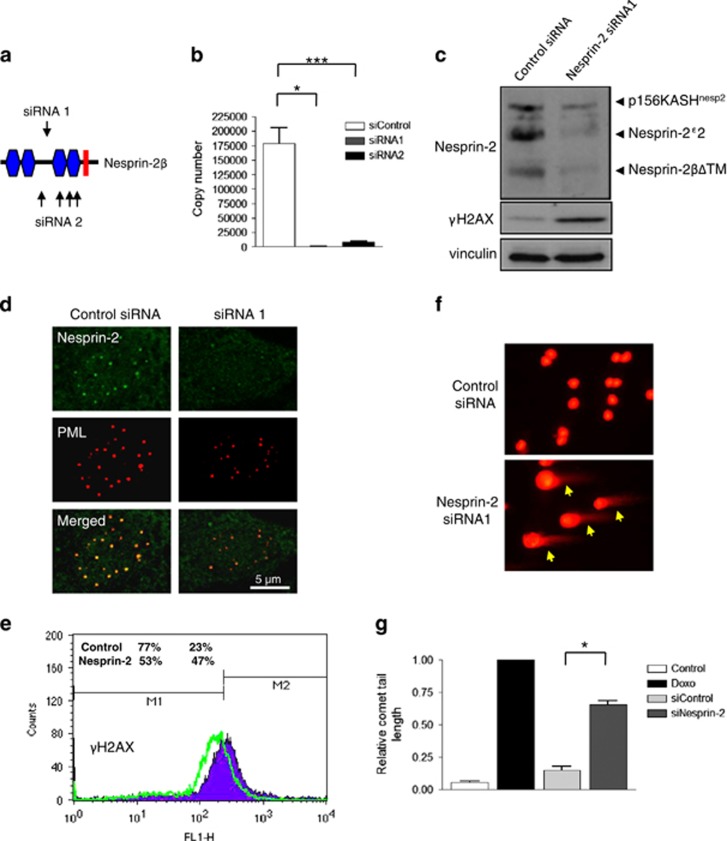
Disruption of nesprin-2 increases basal levels of DNA damage. (**a**) Schematic representation of the siRNA oligomer positions targeting nesprin-2*β*ΔTM. (**b**) qPCR confirmed efficient knockdown of the nesprin-2*β*ΔTM 5'UTR using two independent strategies; a single siRNA (siRNA 1) or a pool of siRNA (siRNA 2). Graph shows the combined data of three independent experiments repeated in triplicate (*P=0.0028 and ***P=0.0038, respectively). (**c**) WB analysis of control and nesprin-2-depleted VSMCs. (**d**) IF showing nesprin-2 (green) and PML (red) localisations in control and nesprin-2-depleted VSMCs. (**e**) FAC analysis of control and nesprin-2-depleted cells displaying *γ*H2AX staining. (**f**) IF of comet assay after control and nesprin-2 depletion. (**g**) Quantification of comet assays (*P=0.0005). Graph represents the combined data from three independent experiments

ERK1/2 enhance ATM/ATR-mediated signalling during DNA repair, so we next examined the effect of releasing ERK1/2 from the complex in presenescent VSMCs by depleting nesprin-2. SiRNA knockdown was confirmed by qRT-PCR and WB (Figures 6a–c), and IF confirmed the loss of nesprin-2 from PML NBs (Figure 6d). WB analysis showed that complex disruption induced the DNA damage shown by increased *γ*H2AX staining by WB and flow cytometry (Figures 6c and e). Comet assays confirmed the presence of unrepaired DNA damage shown by significantly increased comet tail intensity in nesprin-2-depleted cells. In contrast, increased DNA damage was not observed in PML-depleted VSMCs, suggesting that nesprin-2 is the key ERK scaffold in the complex (Figures 6f and g, and Supplementary Figure 10).

**Figure 7:**
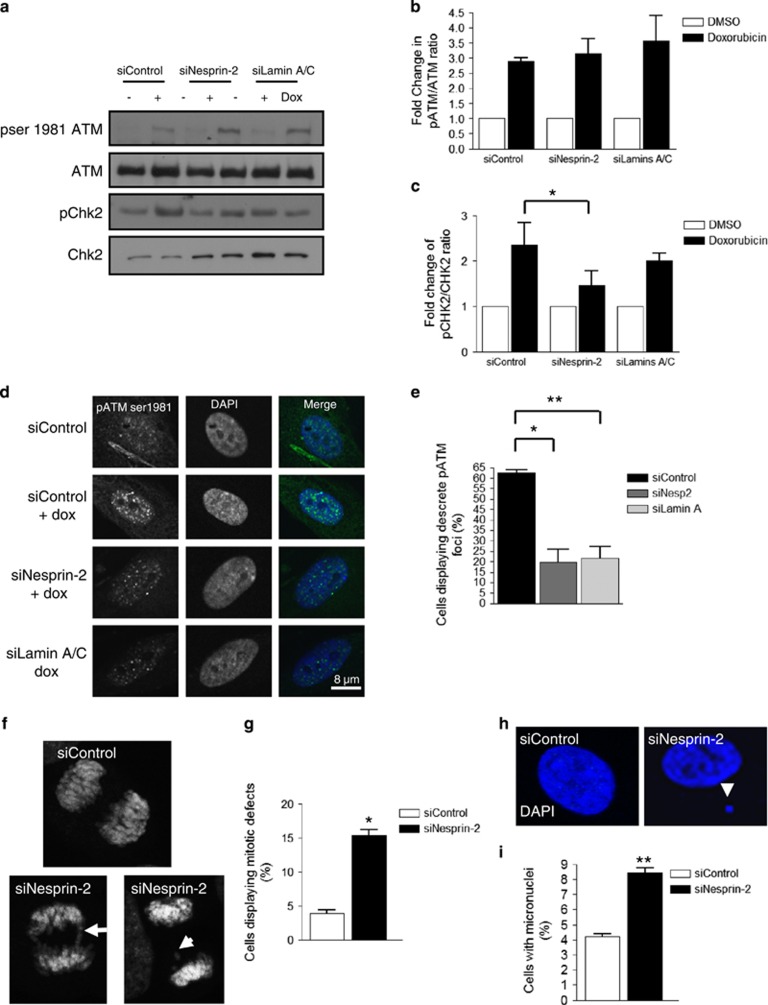
Nesprin-2 regulates ATM spatial organisation and signalling fidelity in response to DNA damage. (**a**) WB analysis of control, nesprin-2- and lamins A/C-depleted VSMCs in the presence of DMSO (control) or doxorubicin for 1 h. (**b**) Graph shows the combined data of three independent experiments of fold change in pSer1981 ATM/total ATM ratio determined by densitometry analysis. (**c**) Graph shows the combined data of three independent experiments of fold change in pCHK2/total CHK2 ratio determined by densitometry analysis (*P=0.0166). (**d**) IF of control, nesprin- and lamins A/C-depleted cells treated for 1 h with doxorubicin; pSer1981 ATM (green) and DAPI (blue). (**e**) Quantification of cells displaying pSer1981 ATM foci after 1-h doxorubicin treatment (*P=0.0270 and **P=0.0179). Graph represents the combined data of three independent experiments counting 300 cells per condition. (**f**) IF showing DAPI staining of mitotic control and nesprin-2-depleted cells showing bridging (arrow) and lagging (arrowhead) chromosomes during mitosis. (**g**) Quantification of nuclei displaying mitotic defects. Graph shows the number of mitotic defects observed in three independent experiments (*P=0.016). (**h**) IF showing DAPI staining of control and nesprin-2-depleted VSMCs. Arrowhead marks micronucleus. (**i**) Quantification of the number of cells displaying micronuclei (**P=0.0025). Graph shows the combined data of three independent experiments counting 300 cells per condition

Next, we sought to determine the signalling pathways within the DDR that regulate ERK compartmentalisation. Depletion of the DDR kinase ATM in VSMCs blocked the dynamic reorganisation of nesprin-2 and PML into nucleolar caps in response to DNA damage (Supplementary Figure 11) suggesting a link between ERK and ATM signalling. To investigate this further, nesprin-2 and lamin A/C-depleted cells were treated with doxorubicin. Doxorubicin treatment stimulated the ATM activity (marked by ser1981 phosphorylation) in control VSMCs, and this response was also observed in nesprin-2 and lamin A/C-depleted VSMCs (Figures 7a and b). However, analysis of the downstream ATM target Chk2 revealed that Chk2 phosphorylation in response to DNA damage was significantly impaired in nesprin-2-depleted, but not lamin A/C-depleted, VSMCs, although lamin A/C-depleted lysates displayed a modest, nonsignificant reduction in Chk2 phosphorylation (Figures 7a and c). Control nuclei displayed multiple ATM foci in response to DNA damage (62.67±2.082; Figures 7d and e), whereas nesprin-2 or lamins A/C depletion blocked the induction of ATM foci in response to DNA damage (19.67±10.69 and 21.67±9.609, respectively; Figures 7d and e) suggesting ERK compartmentalisation during the DDR is also essential for maintaining ATM localisation and activation of downstream ATM targets in the presence of DNA damage.

In addition to their role in regulating DNA repair, ATM/ATR also activate cell cycle checkpoints. Nesprin-2-depleted VSMCs displayed a fourfold increase in cells with mitotic defects, such as telophase bridges and lagging chromosomes (Figures 7f and g), as well as a twofold increase in cells displaying micronuclei (Figures 7h and i) indicative of G2/M checkpoint failure and genomic instability.

## Discussion

### Prelamin A accumulation promotes ERK compartmentalisation and VSMC senescence

In this study, we show that VSMC ageing *in vitro* is characterized by three distinct growth phases: rapid proliferation in young cells is followed by a presenescent phase characterised by a slowing of cell growth and the accumulation of prelamin A, which is then rapidly followed by growth arrest and cellular senescence. Prelamin A accumulation in presenescent VSMCs was associated with increased DNA damage and also with increased compartmentalisation of ERK1/2 at PML NBs, suggesting that signals associated with prelamin A accumulation and/or DNA damage may induce complex formation.^2, 7^ The consistent accumulation of prelamin A in presenescent VSMCs suggests that prelamin A may have unique functions in the DDR in VSMCs. We speculate that prelamin A may be part of a feedback mechanism to block VSMC proliferation, as low levels were associated with nuclear reorganization of signalling complexes, which promoted cell cycle delay and DNA repair. However, when DNA damage persists, prelamin A accumulates further and can interfere with DNA damage repair ultimately leading to robust cellular senescence^2, 3^ as observed in VSMCs and Zmpste24/FACE1-deficient fibroblasts.^19^ Prelamin A accumulation in proliferative VSMCs or SMCs derived from HGPS iPS cells has also been shown to lead to mitotic failure.^20^ This cellular outcome is likely to further accelerate senescence and may contribute to the rapid induction of senescence following prelamin A accumulation. Clearly, further elucidation of the factors that regulate prelamin A accumulation in VSMCs during the DDR and VSMC ageing are now required.

### Lamin A associates with nesprin-2, ERK and PML

Lamins A/C form a filamentous network throughout the nucleus that spatially organises DNA-repair and nuclear signalling events.^6^ We speculated that lamins A/C were good candidates to spatially regulate the nesprin-2/ERK/PML complex, and we confirmed that this complex interacts with the C-terminus of lamin A. Nesprin-2 and ERK1/2 have previously been shown to independently interact with lamin A; nesprin-2 binds to the lamin A tail via its SRs, whereas ERK1/2 bind to coil 2 of lamin A, suggesting that nesprin-2*β*ΔKASH1 also couples ERK1/2 to the lamin A tail, independently of the coil 2/ERK interaction.^21, 22, 23^ Moreover, lamins A/C disruption resulted in increased PML NB size in VSMCs, and this is consistent with the larger PML NBs with reduced mobility observed in lamin A-deficient nuclei.^18^ Changes in PML size were also observed in VSMCs with prelamin A accumulation; however, whether these larger bodies have different functionality has not been determined. Lamins A/C disruption also resulted in disruption of ERK compartmentalisation, suggesting that lamins A/C perform a scaffolding role anchoring the nesprin-2/ERK complex at PML NBs during the DDR. However, despite the involvement of lamins A/C in nesprin-2/ERK compartmentalisation, lamins A/C depletion did not impair, ATM or Chk2 phosphorylation and DNA damage continued to be efficiently repaired. This suggests that lamins A/C and ERK compartmentalisation are not essential for DNA repair and that nesprin-2/ERK maintains signalling integrity even when diffusely localised in the nucleoplasm.

### Efficient DNA repair does not require PML NBs in VSMCs

Although PML NBs have been implicated in DNA repair, their importance remains controversial.^13, 24^ Numerous DNA-repair proteins, including ATM and ATR, are known to localise at PML NBs in the absence of DNA damage.^25, 26, 27^ Genotoxic stress increases the PML NB number, stimulates their association at DNA lesions and induces the loss of these repair proteins from PML NBs.^25, 26, 27^ Post-translational modification is proposed to play a role in the relocalisation of these proteins raising the intriguing possibility that ERK1/2 recruitment to PML NBs may stimulate the redistribution of repair proteins. In addition, PML NBs may serve to store active ERK1/2 during the DDR, and further investigation is required to clarify the relationship between PML NBs and ERK1/2 signalling in the nucleus. DNA damage also stimulated organisation of PML-associated ERK1/2 into nucleolar caps. The function of these nucleolar caps within the DDR has yet to be fully elucidated, although PML has been shown to influence p53 activity by sequestering MDM2 at the nucleolus.^14, 15, 28^ Thus, nucleolar caps may serve as hubs to regulate the cell cycle during the DDR. However, despite these connections between PML NBs and DNA repair, we show that PML disruption did not ablate DNA repair in VSMCs (Supplementary Figure 5). This observation is consistent with a previous study demonstrating that PML NBs are nonessential for DNA repair and senescence in fibroblasts.^29^ Importantly, depletion of lamins A/C or PML disrupted ERK2 compartmentalisation, suggesting that both serve as scaffolds to assist lesion repair. However, disrupted ERK compartmentalisation, either by lamins A/C or PML depletion, failed to ablate DNA repair, further suggesting that nesprin-2*β*ΔKASH1 remains associated with ERK1/2 and signalling fidelity remains intact even when compartmentalisation is lost.

### Nesprin-2*β*ΔKASH1 is essential for efficient DNA repair and cell cycle delay

In contrast to lamins A/C and PML, nesprin-2*β*ΔKASH1 was essential for efficient DNA repair in VSMCs. We have previously shown that nesprin-2*β*ΔKASH1 depletion stimulated VSMC proliferation, suggesting that this complex may regulate cell cycle progression and in support of this notion, nesprin-2 depleted VSMCs displayed mitotic defects and micronuclei formation, indicative of a G2/M checkpoint defect.^10, 16^ Although previous studies have implicated ERK1/2 in regulating cell cycle progression in response to DNA damage,^16, 30^ for the first time we implicate nesprin-2 in the G2/M checkpoint. The failure of nesprin-2 depleted cells to block mitotic entry may be twofold; due to a failure of ERK1/2 to phosphorylate target proteins that are required to activate G2/M arrest and/or due to the activation of inappropriate ERK target proteins that promote cell cycle progression. However, despite the existence of a large body of evidence implicating ERK1/2 in DNA repair and the DDR, our knowledge of the proteins ERK1/2 target during this process is lacking, although ATM/ATR are possible candidates as they also regulate activation of cell cycle checkpoints in response to DNA damage.

Nesprin-2*β*ΔKASH1 is comprised of two sets of double SRs separated by a flexible unstructured linker region and although our knowledge of nesprin-2*β*ΔKASH1-binding partners remains limited, we have previously shown that phosphorylated ERK1/2 can bind to the N-terminal SRs of nesprin-2*β*ΔKASH1.^10, 31, 32^ An intriguing possibility is that nesprin-2*β*ΔKASH1 may also interact with ERK1/2 target proteins involved in DNA repair. Owing to the flexibility between the N-terminal and C-terminal SR modules, nesprin-2*β*ΔKASH1 may enhance the phosphorylation of ERK1/2 targets by bringing substrates into close proximity with ERK1/2.^31, 32^ Indeed, other ERK1/2 scaffold proteins, such as MEK-partner-1 and kinase suppressor of RAS, are known to interact with several components of the RAS/RAF/MEK/ERK pathway to enhance signalling.^33, 34, 35^ Further studies focused on identifying novel nesprin-2*β*ΔKASH1-binding partners, particularly those involved in DNA repair, are required to delineate the precise roles of both nesprin-2*β*ΔKASH1 and ERK1/2 in the DNA-repair process.

## Materials and Methods

### Cell culture

Human aortic VSMCs from four different male and female donors ranging in age from 20 to 54 years were cultured as described previously, and characterised for proliferation capacity and senescence. For all studies, excluding those requiring serial passaging to presenescence and senescence, VSMCs in the proliferative phase (P6–P9) were used.^36^ Nesprin-2 siRNA oligomers targeting the nesprin-2*β*ΔKASH1 variant have been described previously.^10^ PDT was determined using the online calculator at www.doubling-time.com/compute.php. VSMCs were plated at a known density and counts were performed on each passage until senescence. ATM, ATR, FACE1 and lamin-A smart pool siRNA oligomers from Dharmacon (Lafayette, CO, USA) were used in this study. Transfection of siRNA was performed using Hyperfect (Qiagen, Venlo, Netherlands), as per the manufacturer's instructions. Cells were treated with 0.5 *μ*M Doxorubicin (Sigma, St. Louis, MO, USA) for the indicated time to induce DNA damage. ATM/ATR and ERK1/2 inhibition was achieved by incubating cells overnight in the presence of 5 *μ*M CGK733 (Sigma) or for the time stated with 20 *μ*M U0126 (Cell Signaling Technologies, Danvers, MA, USA), respectively.

### Antibodies, IF and flow cytometry

Antibodies used for WB, IF and IPs were the following: ATM (ab78), ATR (ab2905), nucleolin (ab13541; Abcam, Cambridge, UK); pH2AX (#9718), pSer15 p53, pSer1981 ATM (#5883), 53BP1 (#4937), pChk2 (T68) (#2661), Chk2 (#6334; Cell Signaling Technologies); Face-1 C-13 (sc-34777), prelamin-A C-20 (sc-6214), lamin A/C N-18 (sc-6215), PML PG-M3 (sc-966), ERK2 D-2 (sc-1647), pERK1/2 E-4 (sc-7383; Santa Cruz, Dallas, TX, USA); Vinculin (V9131), *β*-actin (A5316; Sigma); and nesprin-2 N3. Secondary antibodies for WB were horseradish peroxidase-conjugated antimouse (NA931) or antirabbit (NA94V) antibodies from GE Healthcare (Lafayette, CO, USA). ECL chemiluminescent kit (RPN2132, GE Healthcare) was used for the detection according to the manufacturer's instructions. Invitrogen (Paisley, UK) Anti-Mouse Alexa Fluor 568 (A11031) and Anti-Rabbit Alexa Fluor 488 (A11034, Invitrogen) were used as IF secondary antibodies. Cells were cultured on cover slips, fixed in 50% methanol/acetone and processed as described previously. SA-*β*-gal staining was performed as described previously.^2^ For observing the DNA content of cells by propidium iodide staining, cells were trypsinised and washed three times in PBS. Cells were fixed in ice-cold 75% ethanol for 30 min at 4 °C. DNA staining was achieved by addition of PI and RNase for 45 min at 37 °C. For *γ*H2AX staining cells were processed as per the manufacturer's instructions (Cell Signaling Technologies).

### Confocal microscopy and data analysis

All images were captured at × 63 magnification using a Leica (Milton Keynes, UK) SP5 laser scanning confocal microscope. PML number and size was measured using the find object function in the Volocity software (PerkinElmer, Waltham, MA, USA).

### IPs and subcellular fractionations

*In vivo* IPs, GST-pull downs and subcellular fractionations were performed as described previously.^10^

### Comet assays

DNA damage was assessed using the Comet assays as described previously.^2^ Protocols were obtained from the Comet Assay Interest Group website (www.cometassay.com).

### Statistics

The results are presented as mean±S.E.M. For the comparison of proliferative/presenescent/senescent groups, unpaired Student's *t*-tests were performed. For siRNA knockdown groups, paired Student's *t*-tests were performed.

## Abbreviations

DDR: DNA damage response
VSMC: vascular smooth muscle cell
ATM: ataxia telangiectasia-mutated protein
ATR: ataxia telangiectasia- and Rad3-related protein
ERK1/2: extracellular signal regulated kinases 1 and 2
PML NB: promyelocytic leukaemia protein nuclear bodies
NE: nuclear envelope
HGPS: Hutchinson Gilford Progeria Syndrome
iPS: induced pluripotent stem cells
SA-*β*-gal: senescence-associated-*β*-galactosidase
WB: Western blot
IF: immunofluorescence microscopy
SR: spectrin repeat
