# Supplementary material for: Nesprin-2-dependent ERK1/2 compartmentalisation regulates the DNA damage response in vascular smooth muscle cell ageing
Source: Cell Death Differ. 2015 Mar 6;22(9):1540–50. doi: 10.1038/cdd.2015.12 (PMC4532777; doi:10.1038/cdd.2015.12)
Supplement: Supplementary Figures [file cdd201512x1.pdf]

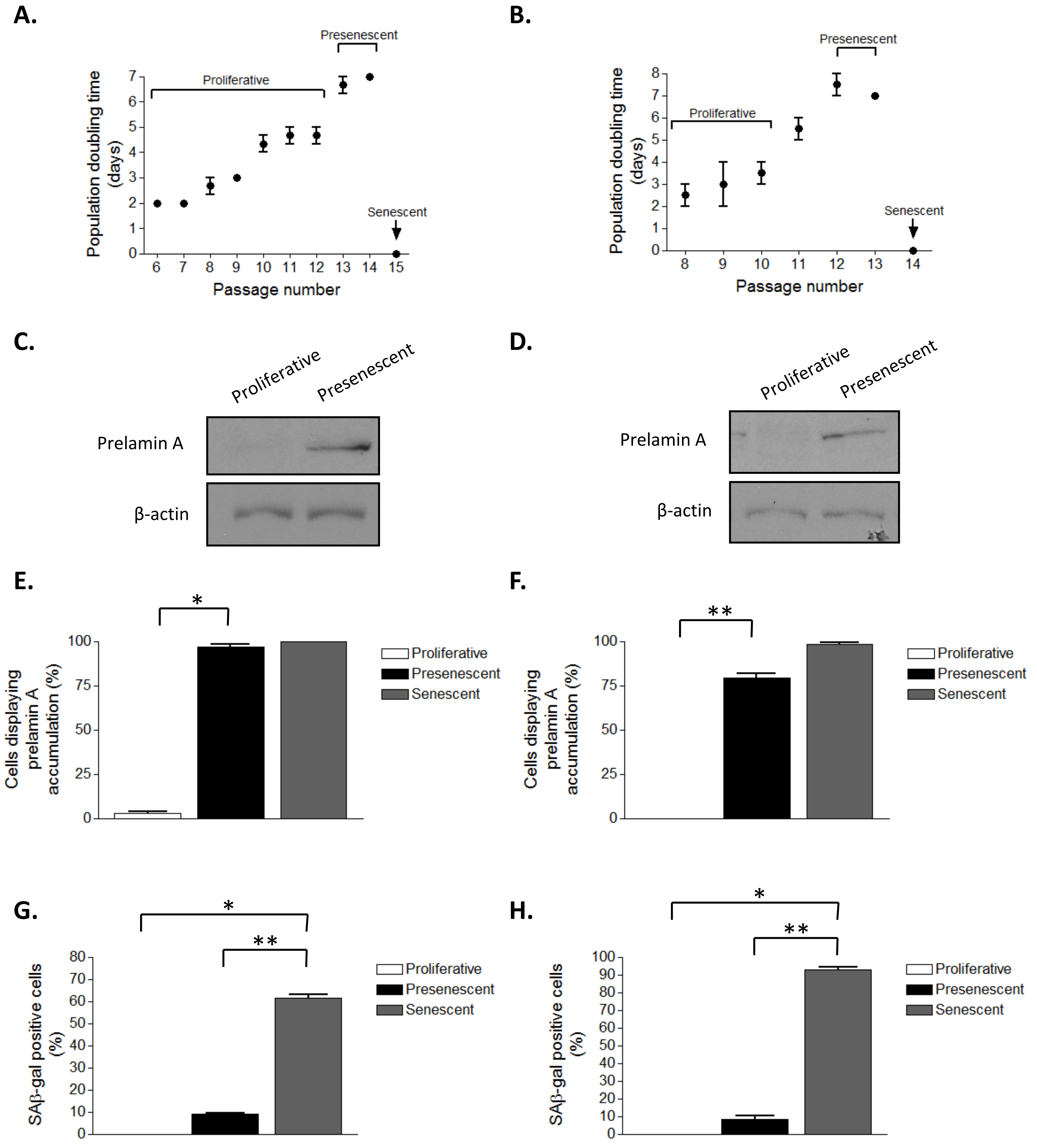

Supplementary Figure 1. Growth curves and prelamin A accumulation of 35F and 52M VSMC lines. PDT of A) 35F and B) 52M VSMCs grown *in vitro*. Each graph shows combined data of 3 independent experiments. WBs showing prelamin A accumulation in C) 35F and D) 52M VSMCs in presenescence. Graphs show combined data of independent experiments counting the percentage of cells displaying prelamin A accumulation determined by IF for E) 35F (*p=<0.0001) and F) 52M (**p=<0.0001) VSMCs. Quantification of percentage of cells staining positive for SA-βgal for G) 35F (*p=<0.0001 and **p=<0.0001) and H) 52M (*p=<0.0001 and **p=<0.0001) VSMCs. Graphs show combined data for 3 independent experiments.

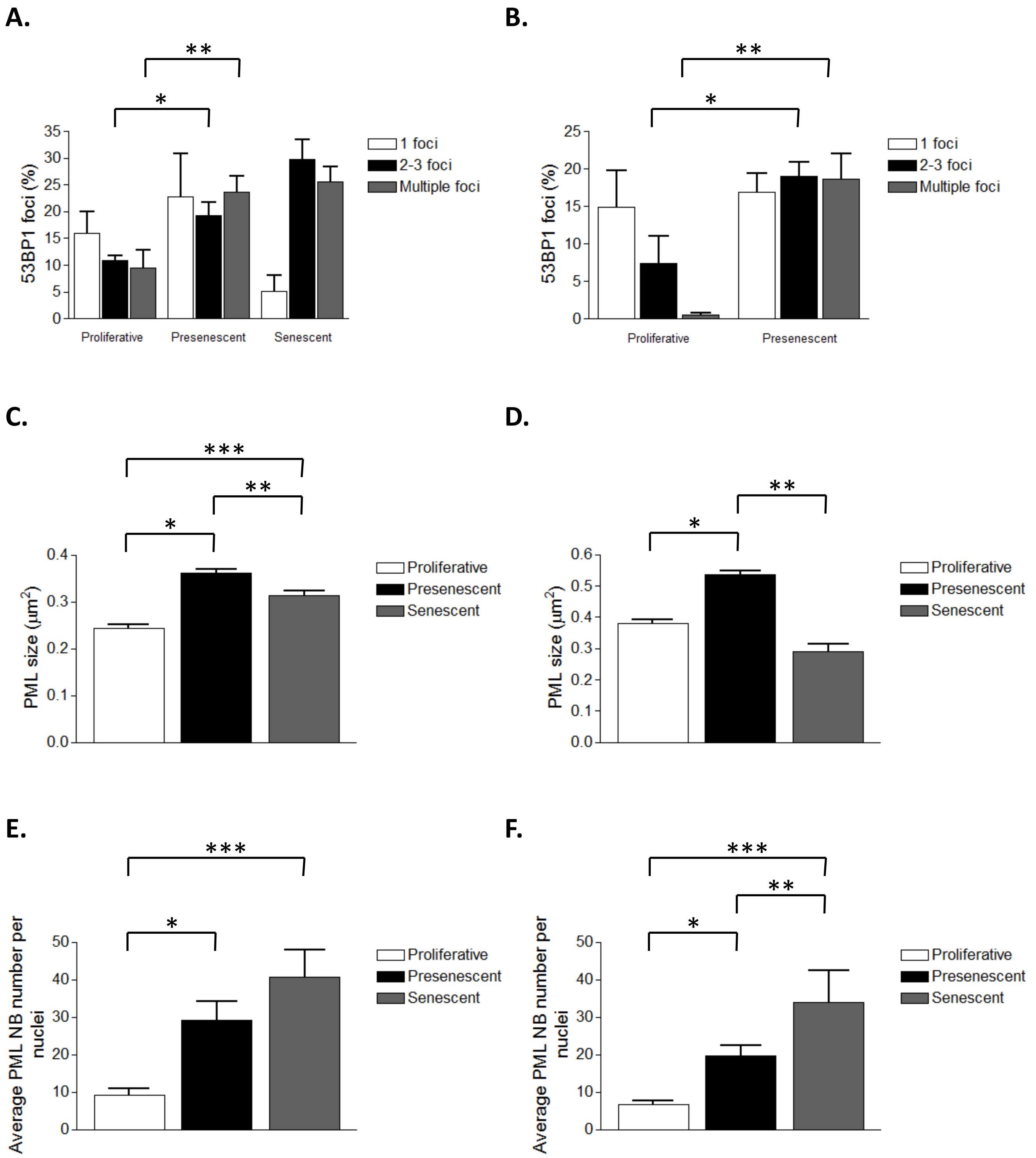

Supplementary Figure 2. 53BP1 foci and PML NB analysis of 35F and 52M VSMC lines. 53BP1 foci analysis for proliferative, presenescent and senescent A) 52M (*p=0.0357 and **p=0.0324) and B) 35F (*p=0.0481 and **p=0.0061) VSMCs. Average PML size in proliferative, presenescent and senescent C) 52M (*p<0.0001, **p<0.0001 and ***p<0.0001) and D) 35F (*p<0.0001, **p<0.0001 and **p=0.05) VSMCs. Average PML number per nuclei in proliferative, presenescent and senescent E) 52M (*p=0.01, **p<0.001) and F) 35F (*p=0.05, **p=0.05 and ***p=0.01) VSMCs. Graphs represent combined data from 3 individual experiments analysing 300 cells.

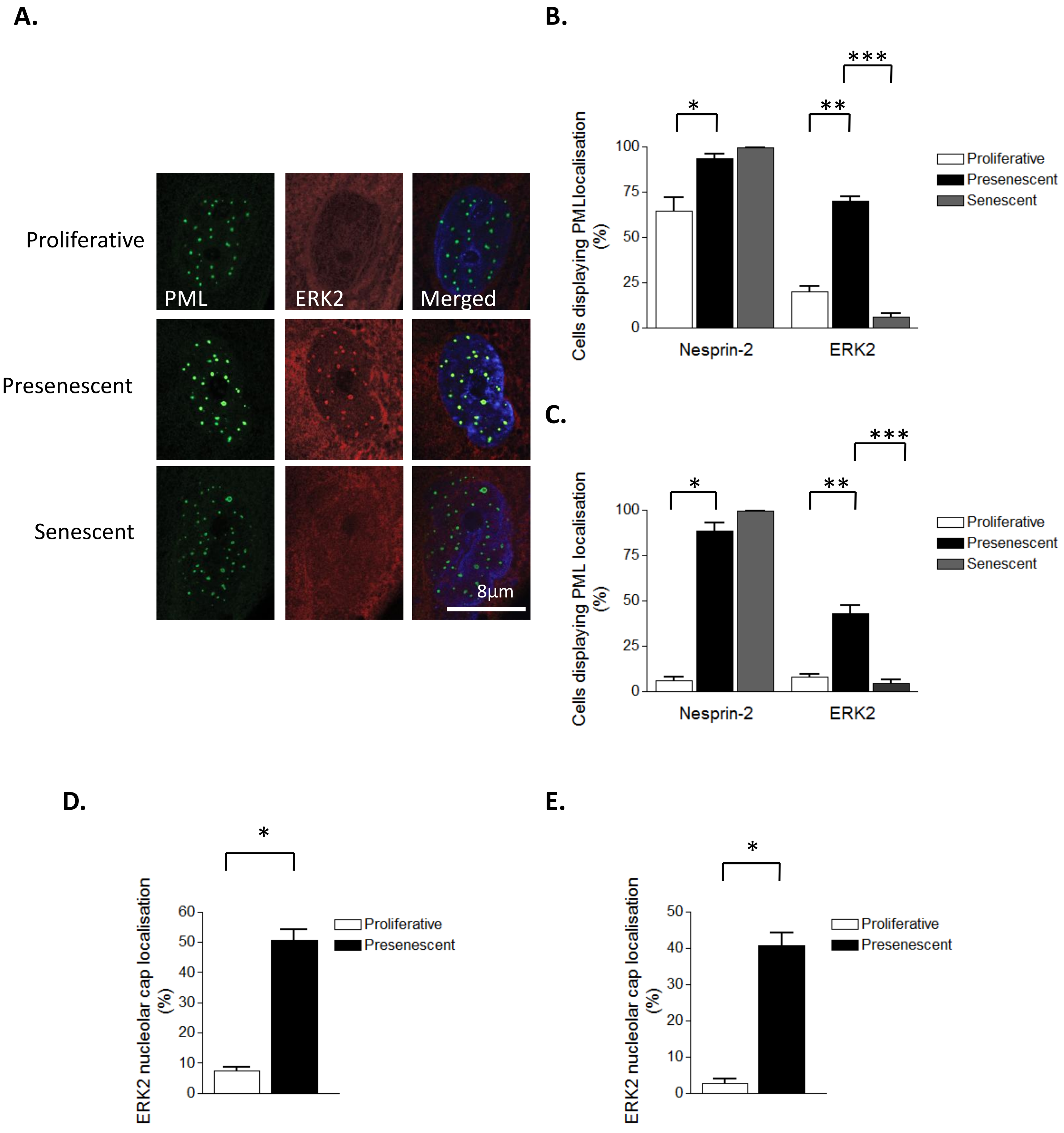

Supplementary Figure 3. A) IF analysis of PML (green), ERK2 (red) and DAPI (blue) localisation in proliferative, presenescent and senescent VSMCs. Quantification of nesprin-2 and ERK2 localisation at PML NBs in different primary VSMC isolates B) 35F (*p=<0.001, **p=<0.001 and ***p=<0.001) and C) 52M (*p=0.0196, **p=0.0003 and ***p=<0.0001). ERK2 localisation at nucleolar caps in D) 35F (*p=0.0003) and E) 52M (*p=<0.0001) VSMCs. Graphs represent the combined data of 3 independent experiments counting 300 cells per condition.

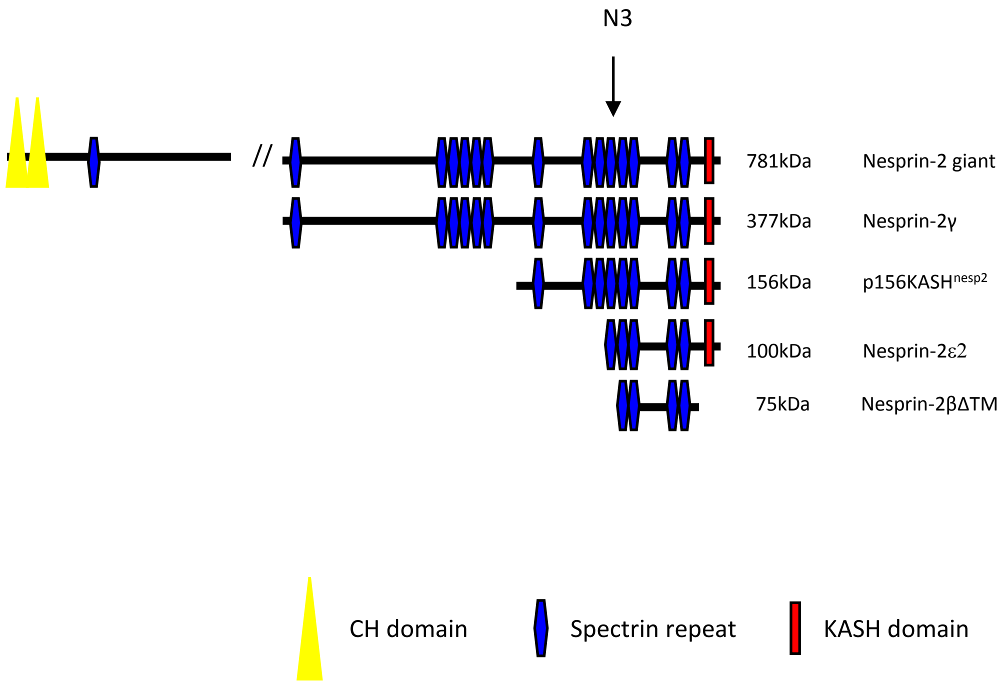

Supplementary Figure 4. Schematic representation of published nesprin-2 isoforms. N3 marks the antibody used in this study.

A.

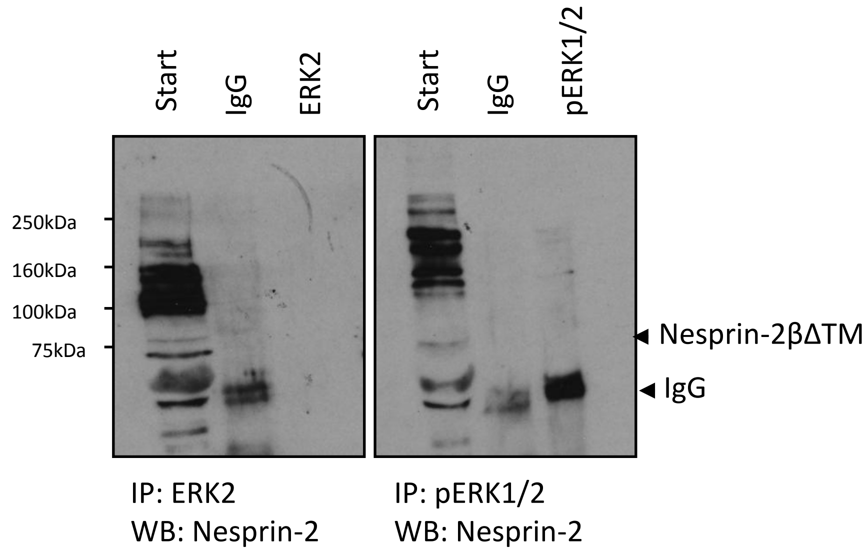

Supplementary Figure 5. Loss of Nesprin-2βΔTM and ERK2 interaction in senescent VSMCs. A) WB analysis of ERK2 and pERK1/2 IPs from senescent VSMCs. Note the lack of nesprin-2βΔTM precipitated by ERK2 and pERK1/2.

A.

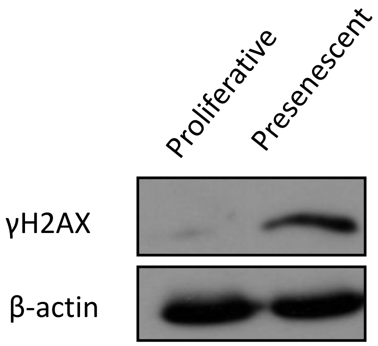

B.

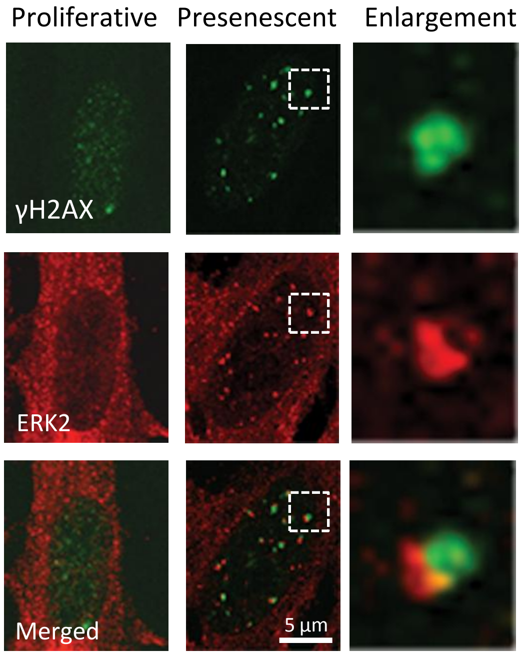

Supplementary Figure 6. ERK2 positive PML NBs abut DNA lesions. A) WB showing γH2AX levels in proliferative and presenescent VSMCs. B) IF showing γH2AX (green) and ERK2 (red) localisation in proliferative and presenescent VSMCs. Enlargement shows ERK2 localises in close proximity to γH2AX lesions in presenescent nuclei.

A.

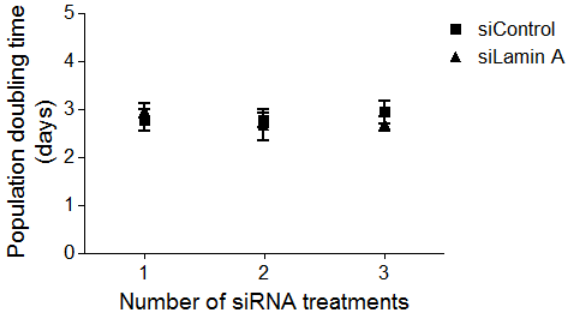

B.

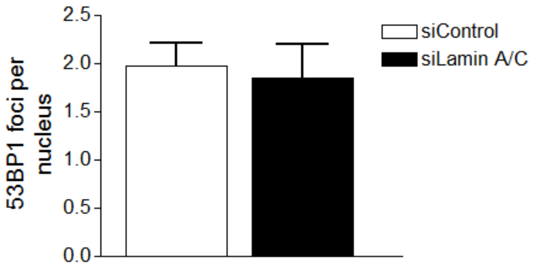

C.

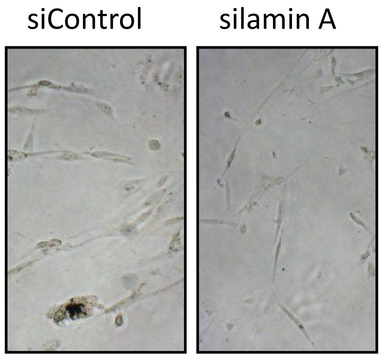

Supplementary Figure 7. Prolonged lamin A/C depletion in proliferative VSMCs does not alter PDT, DNA damage response or senescence. A) PDT of control and lamin A/C depleted VSMCs after 3 consecutive rounds of knockdown. Graph shows combined data from 3 individual experiments. B) Average number of 53BP1 foci per in control and lamin A/C nuclei after 3 consecutive rounds of knockdown. C) Images of control and lamin A/C depleted cells stained for SA-β-gal after 3 consecutive rounds of knockdown.

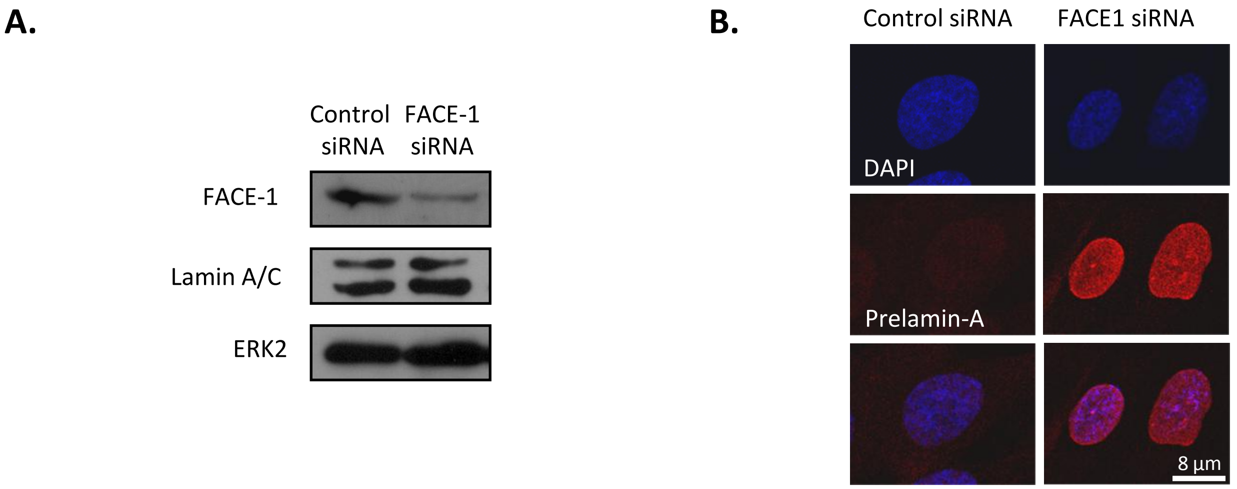

Supplementary Figure 8. Validation of the FACE1 depletion strategy. A) WB confirming FACE1 depletion and B) IF confirming prelamin A accumulation in FACE1 depleted VSMCs.

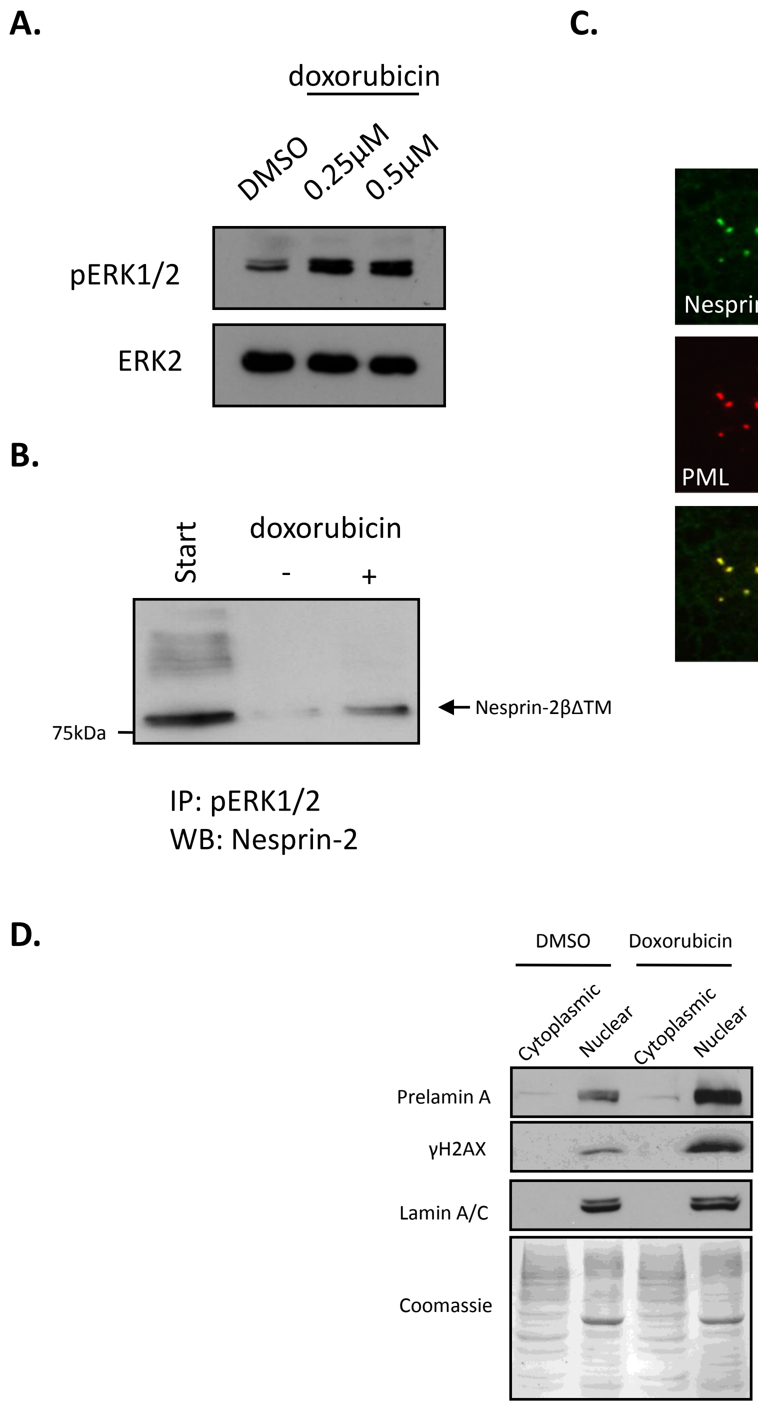

Supplementary Figure 9. DNA damage stimulates nesprin-2/ERK association . A) WB of control and doxorubicin treated VSMCs for pERK1/2 and total ERK2. B) IP of pERK1/2 antibody to pellet the 75kDa nesprin-2βΔKASH1 variant from control and doxorubicin treated VSMCs. C) IF showing nesprin-2 (green) and PML (red) of control and doxorubicin treated VSMCs. Arrowheads mark the nucleolar cap. Nucleolar caps were confirmed by staining for nesprin-2 (green) and the nucleolar marker nucleolin (red). Arrowheads mark nucleolar caps. D) WB of cytoplasmic and nuclear fractions of control and doxorubicin treated (3hr) VSMCs.

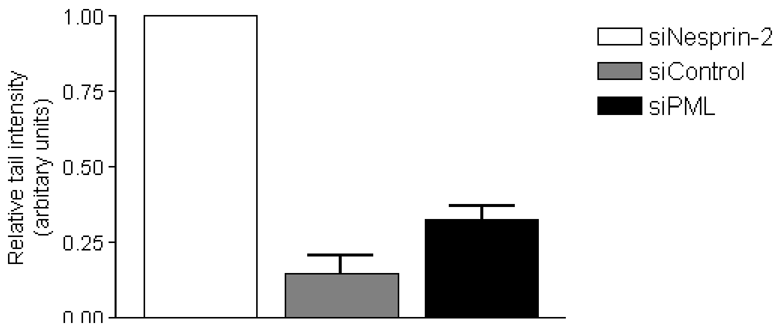

Supplementary Figure 10. PML depletion does not induce DNA damage in VSMCs. Quantification of comet tail length of control, nesprin-2 and PML depleted VSMCs.

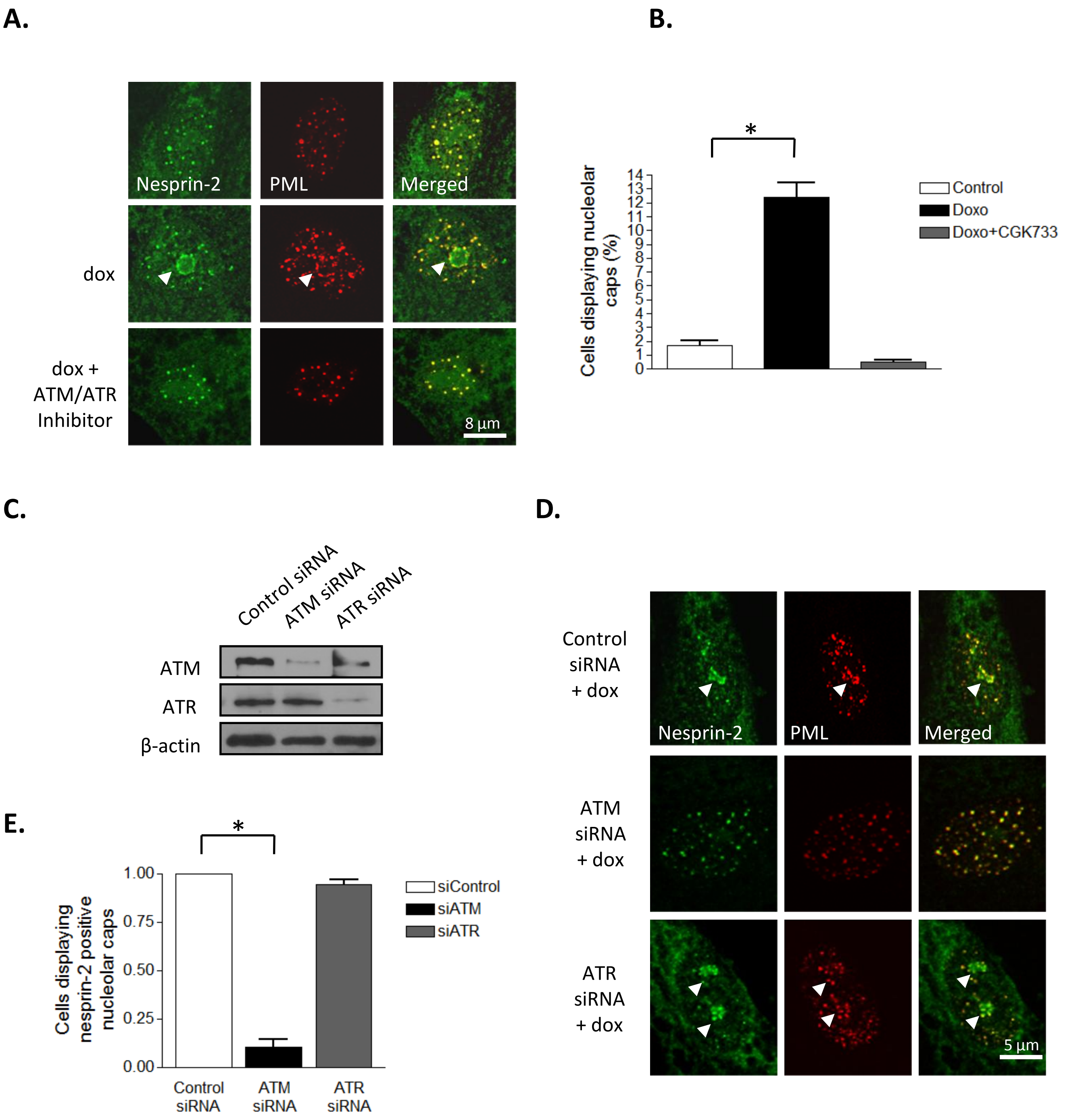

Supplementary Figure 11. Formation of nesprin-2/PML nucleolar caps is ATM dependent in VSMCs. Cells were preincubated with either DMSO or the ATM/ATR inhibitor CGK733 for 2 hours prior to doxorubicin treatment. A) IF microscopy showing nesprin-2 (green) and PML (red). Arrowheads mark nucleolar caps. B) Quantification of nesprin-2/PML nucleolar caps in doxorubicin treated control and inhibitor treated VSMCs (*p = 0.0005). Graph shows combined data of 3 independent experiments counting 300 cells. C) WB confirmed ATM and ATR knockdown. D) IF showing nesprin-2 (green) and PML (red). E) Quantification of nesprin-2 positive nucleolar caps in doxorubicin treated control, ATM and ATR depleted VSMCs (*p = 0.0006). Graph shows combined data of 3 independent experiments counting 300 cells.
